# A rare case of hepatic sub capsular biloma after open cholecystectomy: a case report

**DOI:** 10.4076/1757-1626-2-7836

**Published:** 2009-09-15

**Authors:** Karim Ibn Majdoub Hassani, El Bachir Benjelloun, Abdelmalek Ousadden, Khalid Mazaz, Khalid Ait Taleb

**Affiliations:** Department of General Surgery, Universitet Hospital Hassan IIFesMorocco

## Abstract

Bilomas are localized collections of bile occurring usually post-operatively from an injured cystic or bile duct while most bilomas collect in the subhepatic space. We describe a rare case of hepatic subcapsular biloma after open cholecystectomy successfully treated by percutaneous drainage.

## Introduction

Bile leak after open or laparoscopic cholecystectomy is usually a result of minor biliary injury, although it can sometimes reveal a major duct injury. It is estimated that biloma originates from the cystic duct in more than 50% of the cases [1]. Hepatic subcapsular bilomais an exceptional complication after cholecystectomy. We describe a rare case of a 28-year-old woman, presented 2 weeks after open partial cholecystectomy with signs of a rightsided chest pain, dyspnea and cough as a result of subcapsular biloma diagnosed by a computed tomography (CT) and, successfully treated by percutaneous drainage. There are only some cases reported in the literature of this complication after laparoscopic cholecystectomy, but according to our knowledge and from an intensive literature research, this is the first case which has been described after an open cholecystectomy.

## Case presentation

**Figure 1.**
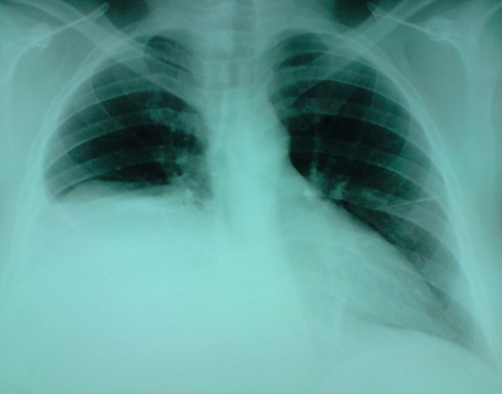
Chest X-ray demonstrated a right pleural effusion.

**Figure 2.**
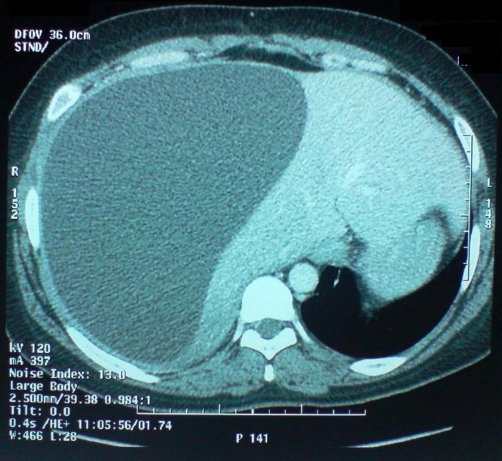
Abdominal CT (axial) demonstrated unilocular subcapsular fluid-filled collection in the right lobe of the liver.

**Figure 3.**
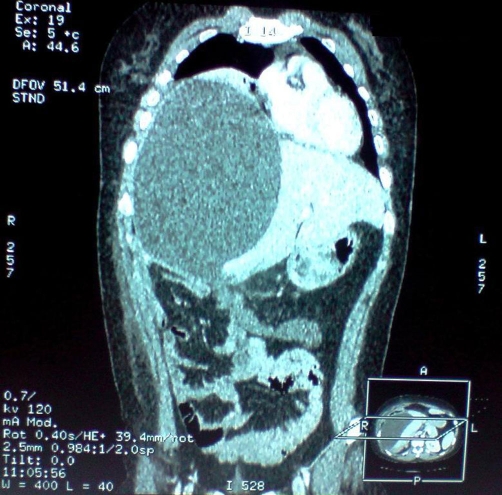
Abdominal CT (coronal) demonstrated unilocular subcapsular fluid-filled collection in the right lobe of the liver.

A 28-year-old previously healthy Moroccan woman presented to the emergency department 15 days after open subtotal cholecystectomy for acute cholecystitis with a 5 days history of rightsided chest pain, dyspnea and cough. She had no abdominal pain, no fever, no vomit and no jaundice. The patient was not using any specific medication and her medical history did not suggest any major disease. She had no prior history of abdominal surgery or trauma. She had a difficult partial cholecystectomy due to severe inflammation in the Calot’s triangle, large empyema in the gall bladder and, excessive bleeding during difficult dissection between gall bladder and liver bed. Subtotal cholecystectomy was done as the cystic duct could not be isolated. The physical examination revealed a conscious woman who’s had a temperature 37 degree C, a pulse rate 90 beats per minute (bpm), a blood pressure 120/70 mm Hg. The abdominal examination revealed a right upper scar without abdominal tenderness. There were no palpable masses or liver enlargement. Laboratory data revealed an hematocrit of 41%, an hemoglobin of 12,6 g/dl, white blood cells of 11,100/mm³ (83% neutrophils), a blood urea of 0,25 g/L, and a creatinine level of 10 mg/L. Liver enzymes showed a total bilirubin of 13 mg/L with a direct component of 6 mg/L; SGOT and SGPT were 87 U/L and 166 U/L, respectively (normal range for SGOT is 17–59 U/L and for SGPT is 21–72 U/L). The chest X-ray showed right-sided chest opacity that simulates pleural effusion (Figure 1). Ultrasonography (US) of the abdomen revealed a large unilocular subcapsular fluid-filled collection in the right lobe of the liver with no free intra-abdominal fluid. Thoraco-abdominal computed tomography (CT) confirmed results of US and concluded a large hepatic sub capsular biloma measuring (21 cm × 16 cm × 14 cm) (Figures 2 and 3). Based on the imaging findings, we made a decision to perform a percutaneous US-guided puncture which removed 2000 ml of bile.

**Figure 4.**
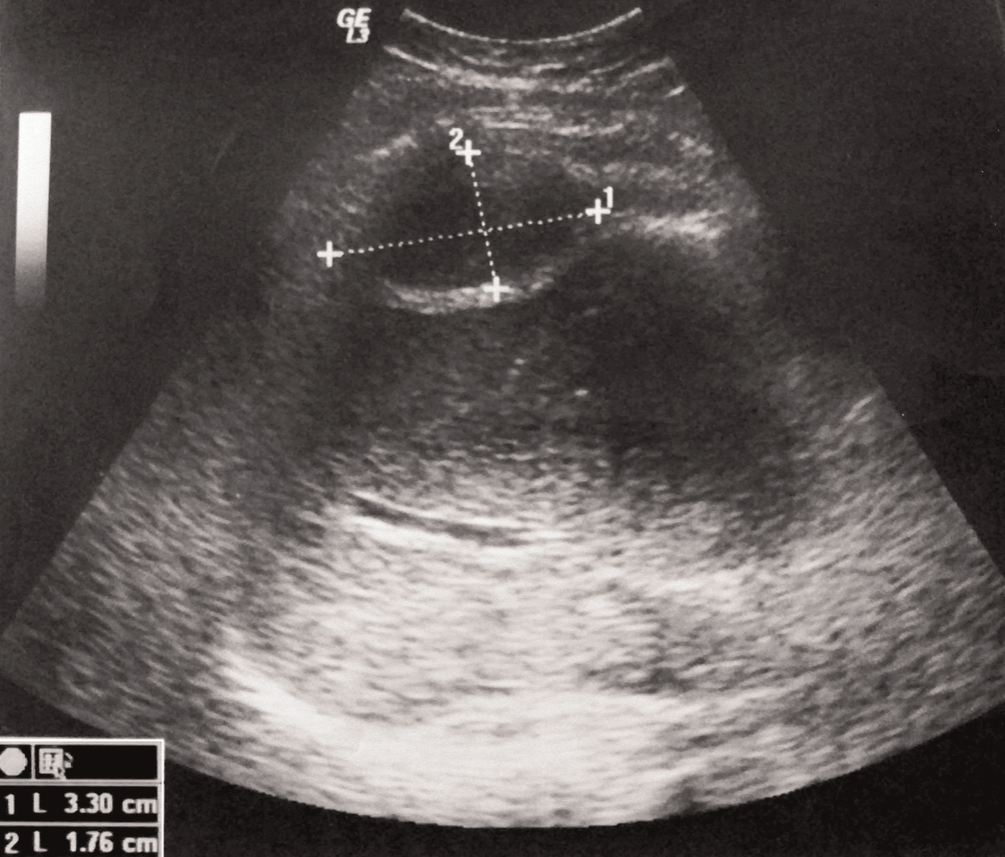
Ultra sonography showed nearly a total vanishing of the biloma, with persistence of a little collection measuring (3.30 × 1.76 cm).

After draining the abscess a drainage catheter was positioned and left in place. The catheter drained more than 500 ml of bile over 5 days and it was removed 2 days later when the output was ceased and, a follow up US showed a near-complete resolution (Figure 4). No complication occurred. We noted that the liver enzymes were normal en days following drainage. The patient was free of any symptoms at the 3 month follow up. A further US, performed after six month later was normal and, did not show any abnormalities.

## Discussion

The term biloma was introduced in 1979 by Gould and Pater to describe a loculated collection located outside the biliary tree. Kuligowska et al. extended the term biloma to include intrahepatic as well as extrahepatic collections of bile. Bilomas mainly result from iatrogenic, traumatic, or spontaneous rupture of the biliary tree [2]. Although bile leakage into the peritoneal cavity is a known complication of open and laparoscopic cholecystectomy [3], the hepatic subcapsular biloma is an exceptional complication after cholecystectomy. Some authors attribute this complication to a small biliary perforated radical because of the backpressure associated with the high-pressure irrigation used during choledochoscopy [4].

High pressure in the proximal biliary ducts, caused by injection of contrast material, is the reported cause of a hepatic subcapsular biloma after ERCP [5]. We think that the possible etiology for the hepatic subcapsular biloma in our patient is a disruption of a small biliary radical near the gallbladder bed during dissection, because the procedure was technically difficult and the anatomy was not well defined. The right upper quadrant abdominal pain is the constant sign in the patient with subcapsular biloma described in the literature, associated sometimes with nausea and vomiting [4]. Our case was unique that the patient presented with respiratory symptoms without any abdominal signs.

Ultrasound is sensitive for diagnosing bilomas, but the diagnosis of this complication is ideally facilitated by the use of Computed tomography [6], Imaging of the biliary tree should be performed early to determine the location and extent of bile leaks [4]. Hepatic subcapsular biloma can be drained percutaneously with removal of the drainage catheter when the output is minimal [4-7]. Our patient and the three other reported cases of bilomas were managed similarly with US guided percutaneous drainage with a good outcome. To conclude a subcapsular biloma is an exceptional complication of cholecystectomy. Early diagnosis and appropriate percutaneous drainage are the key to manage this rarity.
